# DeMambaNet: Deformable Convolution and Mamba Integration Network for High-Precision Segmentation of Ambiguously Defined Dental Radicular Boundaries

**DOI:** 10.3390/s24144748

**Published:** 2024-07-22

**Authors:** Binfeng Zou, Xingru Huang, Yitao Jiang, Kai Jin, Yaoqi Sun

**Affiliations:** 1 School of Automation, Hangzhou Dianzi University, Hangzhou 310018, China; zbf@hdu.edu.cn (B.Z.); xingru.huang@hdu.edu.cn (X.H.); 23320123@hdu.edu.cn (Y.J.); 2 The Second Affiliated Hospital, School of Medicine, Zhejiang University, Hangzhou 310027, China; 3 Lishui Institute, Hangzhou Dianzi University, Lishui 323000, China

**Keywords:** medical image processing, dental X-ray image, segmentation, Mamba, SSM, DCN, deformable

## Abstract

The incorporation of automatic segmentation methodologies into dental X-ray images refined the paradigms of clinical diagnostics and therapeutic planning by facilitating meticulous, pixel-level articulation of both dental structures and proximate tissues. This underpins the pillars of early pathological detection and meticulous disease progression monitoring. Nonetheless, conventional segmentation frameworks often encounter significant setbacks attributable to the intrinsic limitations of X-ray imaging, including compromised image fidelity, obscured delineation of structural boundaries, and the intricate anatomical structures of dental constituents such as pulp, enamel, and dentin. To surmount these impediments, we propose the Deformable Convolution and Mamba Integration Network, an innovative 2D dental X-ray image segmentation architecture, which amalgamates a Coalescent Structural Deformable Encoder, a Cognitively-Optimized Semantic Enhance Module, and a Hierarchical Convergence Decoder. Collectively, these components bolster the management of multi-scale global features, fortify the stability of feature representation, and refine the amalgamation of feature vectors. A comparative assessment against 14 baselines underscores its efficacy, registering a 0.95% enhancement in the Dice Coefficient and a diminution of the 95th percentile Hausdorff Distance to 7.494.

## 1. Introduction

Dental diseases, encompassing periodontal afflictions and caries, are not only confined to oral complications but also implicate broader systemic health ramifications. Numerous studies have corroborated the significant correlation between such dental conditions and elevated risks of cardiovascular diseases, including coronary artery disease, myocardial infarction, and cerebrovascular incidents like strokes [1,2]. Moreover, these conditions are associated with an increased likelihood of ischemic and hemorrhagic strokes as well as cerebral ischemia. The precision of tooth segmentation is crucial in guiding clinical diagnostics and surgical planning. During orthodontic treatments, dentists must monitor tooth movement and root resorption to assess the health status of teeth and correct malocclusions, thus reducing treatment durations [3]. Accurate segmentation from panoramic dental X-ray images is fundamental in this process. The clinical significance of tooth segmentation extends to early diagnostics, enabling the monitoring of progressive dental conditions and assisting in treatment planning. It facilitates the detection of caries, periodontal disease, and developmental anomalies [4]. High-precision, quantitative segmentation methods are thus an essential clinical requirement for preventing and diagnosing dental conditions.

Advancements in automated medical image diagnosis leveraging neural networks for extensive medical image data analysis continue to evolve [5,6]. However, applying these methods to panoramic dental X-ray image segmentation presents numerous challenges, primarily due to the fundamental principles of X-ray imaging, the anatomical and biophysical characteristics of human teeth, and the imaging process itself [7]. X-ray imaging captures images by exploiting the absorption differences when X-rays penetrate materials of varying densities and atomic numbers, creating a two-dimensional representation of three-dimensional tooth structures. The similarity in density and composition of teeth (including dentin and enamel) and adjacent structures (such as gums and alveolar bones), especially when tooth roots meet the jawbone, results in ambiguous boundaries in these regions. Anatomically, the high variability in individual tooth anatomy and the complexity of the root canal system further complicate image processing. From a biophysical perspective, the similarities in X-ray density between teeth and alveolar bones lead to blurred boundaries due to X-rays’ high penetrative and scattering effects. In addition, the projective nature of X-ray images projects three-dimensional structures into a two-dimensional plane, leading to overlaps and intersections that are particularly challenging to discern, especially in multi-rooted, curved teeth. As X-rays penetrate dense tooth and bone regions, scattering effects can also increase background noise and decrease image contrast, further complicating boundary definition. Various artifacts such as scattering, halos, and obstructions may occur during the imaging process, along with imaging equipment limitations and patient movement, which can introduce image noise, thereby diminishing the overall image quality and clarity of dental and surrounding structures.

To augment the fidelity of boundary delineation in panoramic dental X-ray imagery, it is crucial to meticulously account for the inherent constraints of X-ray imaging alongside the anatomical and physiological idiosyncrasies of dental structures and their distinct biophysical attributes. Enhancement of image processing algorithms, especially in regions proximal to tooth roots, necessitates the deployment of models that can a priori emulate dental configurations, concentrating on pivotal areas while conforming to the complex geometries of teeth and their roots. In response to these exigencies, we propose an innovative architecture, termed Deformable Convolution and Mamba Integration Network (DeMambaNet), engineered to address the challenges of segmenting ambiguously defined dental radicular boundaries in panoramic dental X-ray images. This architecture is fortified with three novel components: the Coalescent Structural Deformable Encoder (CSDE), the Cognitively Optimized Semantic Enhancement Module (SEM), and the Hierarchical Convergence Decoder (HCD). The CSDE amalgamates the Deformable Convolution Network (DCN) with the State Space Model (SSM) to harvest multi-scale features and manage spatial dependencies over extended distances. Concurrently, the SEM refines feature representation through sophisticated fusion and encoding strategies, whereas the HCD seamlessly amalgamates features across multiple dimensions, facilitating meticulous detail enhancement from macroscopic to microscopic scales.

The principal contributions of this study are outlined as follows:

1. Proposd DeMambaNet for panoramic dental X-ray segmentation, incorporating a dual-pathway encoder capable of multilevel feature extraction, to address challenges such as the density concordance between dental and osseous tissues, intricate root geometries, and dental overlaps evident. The source code is available on GitHub (https://github.com/IMOP-lab/DeMambaNet) to catalyze expansive research and clinical adoption.
2. Proposed HCD for stratified feature fusion, orchestrate and equilibrate local and global information. Maintaining diversity in feature representation through the Triplet Attentional Feature Integration (TAFI) module across various decoding phases.
3. Implementation of Deformable Convolution and State Space Models to enhance proficiency in managing the compression-induced overlaps and intersections of three-dimensional dental structures into two-dimensional representations through dynamic adaptability of DCN and the spatial resolution capabilities of SSM.

Section 2 discusses the related research, Section 3 elaborates on the methods used, and Section 4 analyzes the results of the proposed approaches. Section 5 describes the discussion, and Section 6 describes the conclusions and future work.

## 2. Related Works

### 2.1. Traditional Computational Approaches in Dental X-ray Segmentation

Segmentation methodologies for dental X-ray imagery have traditionally employed diverse computational strategies to augment diagnostic accuracy. Initially, systems such as fuzzy inference, Bayesian classifiers, and Support Vector Machines were predominant in dental imaging segmentation tasks [8]. These methodologies often necessitated manual expert input to generate precise rule sets, which presented substantial barriers to scalability and utility in routine clinical applications.

### 2.2. Advancements in CNN-Based Dental Image Segmentation

Recent advancements have seen a paradigm shift towards deep learning technologies, particularly by integrating Convolutional Neural Networks (CNNs) for the semantic segmentation of panoramic dental X-ray images [9,10,11]. This transition facilitates enhanced feature extraction capabilities and superior classification accuracy of dental anomalies. Additionally, Buhari et al. [12] have amalgamated fuzzy C-means clustering and level set methods with sophisticated frameworks such as Faster R-CNN and YOLO V5 to tackle the complexities of dental image segmentation and caries detection, thereby illustrating the potent capabilities of deep learning in managing intricate image structures.

A notable advancement within this realm is the adoption of U-Net-based architectures, significantly improving medical image segmentation through refined skip connections and deep supervision techniques. These architectures efficiently harness multi-scale features and integrate sophisticated attention mechanisms [13,14], thus demonstrating substantial promise in dental image segmentation tasks [15]. However, these methods are inherently susceptible to variations in image quality, which may influence diagnostic results, particularly in scenarios involving overlapping dental structures.

### 2.3. Exploration of State Space Models in Image Segmentation

Concurrently with CNN advancements, innovative methodologies like the SegMamba [16] approach have adopted state space models for spatial feature analysis, incorporating tri-oriented spatial Mamba blocks for comprehensive and multi-scale feature representation. This strategy has demonstrated considerable efficacy in precise segmentation prediction by amalgamating multi-scale global insights through a convolutional 3D decoder and by employing skip connections for enhanced feature preservation.

### 2.4. Advancements in Image Segmentation with Deformable Convolutions

The recent InternImage [17] backbone has revolutionized the integration of deformable convolution networks, offering substantial improvements in handling image segmentation and object detection tasks. This model sets a new standard by achieving remarkable performance metrics on challenging datasets like ImageNet and COCO, thus bridging the gap between traditional CNNs and Vision Transformers.

### 2.5. Feature Enhancement and Fusion Techniques for Improved Segmentation

Efficient feature enhancement and fusion methods such as Efficient Vision Center (EVC) [18] and Attentional Feature Fusion (AFF) [19] are widely applied to the computer vision field. The EVC utilizes a dual approach: a lightweight Multi-Layer Perceptron (MLP) captures global long-range dependencies while a vision centre mechanism preserves local details. These are concatenated to form an enriched feature map that effectively balances global and local information. In feature fusion, the AFF method furthers the integration process by employing a multi-scale channel attention mechanism. This mechanism refines the feature maps by selectively weighting features through learned attention weights, thus optimizing the feature integration across different scales.

These methodologies collectively underscore a transition from traditional rule-based systems to more sophisticated, data-driven approaches in dental image segmentation, highlighting the importance of advanced machine learning techniques in improving diagnostic accuracy and operational efficiency in dental care.

## 3. Methods

The primary challenges arise from the intricacies of radiographic imaging techniques, the complex anatomical structure of teeth and their biophysical properties. Firstly, X-ray imaging relies on the differential absorption rates of X-rays by various materials, but the density and atomic numbers of dentin, enamel, surrounding alveolar bone, and gums are closely matched. This similarity is particularly pronounced at the interfaces between tooth roots and the jawbone, leading to reduced image contrast and complicating the boundary detection. From an image processing perspective, the projective nature of X-ray images means that the three-dimensional structure of teeth appears overlapped and intertwined in two dimensions, posing significant challenges, especially with curved multi-rooted teeth. Additionally, the scattering of X-rays in areas of high density introduces noise, degrading image quality. These factors collectively hinder traditional image processing techniques from accurately delineating different dental structures.

**Figure 1:**
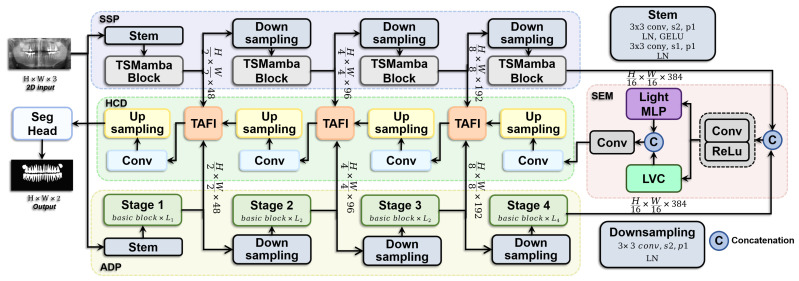
Schematic representation of the Deformable Convolution and Mamba Integration Network (DeMambaNet), integrating a Coalescent Structural Deformable Encoder, a Cognitively-Optimized Semantic Enhance Module, and a Hierarchical Convergence Decoder.

To address these challenges, as depicted in Figure 1, we propose the “Deformable Convolution and Mamba Integration Network” model, a novel 2D dental X-ray image segmentation framework that leverages a dual-pathway encoder structure incorporating DCN and SSM. This model features a Coalescent Structural Deformable Encoder that exploits the distinct characteristics of each pathway, combining DCN and SSM to extract multi-scale features and manage global long-range dependencies. The cognitively-optimized Semantic Enhance Module also integrates and enhances feature outputs, balancing local detail and global information representation through an efficient coding strategy and architectural optimizations. Finally, the Hierarchical Convergence Decoder dynamically fuses features across multiple scales to ensure detailed processing from coarse to fine resolutions. Each of these modules, CSDE, SEM, and HCD, is designed to address the specific complexities encountered in dental X-ray image segmentation, with subsequent sections discussing their design principles and functionalities in detail.

### 3.1. Coalescent Structural Deformable Encoder (CSDE)

**Figure 2:**
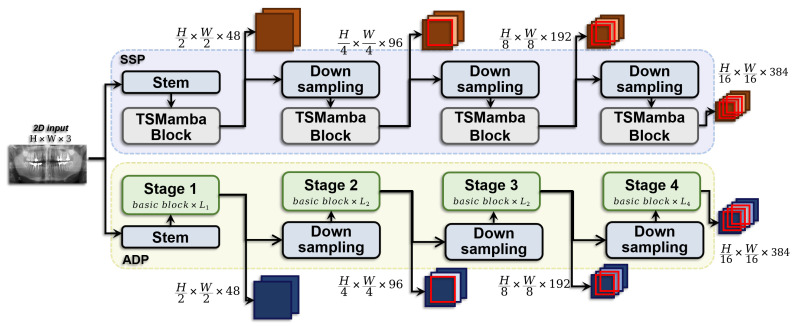
Schematic representation of the CSDE, which integrates a State Space Pathway, based on SSM, in the upper section, and an Adaptive Deformable Pathway, based on DCN, in the lower section.

We employ a dual-pathway encoding strategy to address the significant challenges presented by dental X-ray imaging, particularly the density similarity between dental tissues and bone, complex root geometries, and overlapping of adjacent tooth structures. This strategy manifests in the CSDE, which coalesces a pathway based on DCN and another grounded in SSM, as illustrated in Figure 2.

The design philosophy governing the parallel coalescence of the encoder’s dual pathways is rooted in their synergistic interplay. It effectively leverages Deformable Convolution Networks’ adaptive feature extraction capabilities alongside the efficient management of long-range dependencies facilitated by the Spatial Semantic Module. This integration strategy enables sophisticated modulations of the convolutional kernels within DCN, thereby dynamically refining feature maps. Simultaneously, the SSM contributes to the generation of precise spatial feature representations. This design paradigm establishes a robust framework for the coherent amalgamation of local and global information streams, adeptly addressing the complex challenges of dental configurations and morphologies, especially in areas of homogeneous density. The architecture is instrumental in achieving comprehensive macroscopic localization and microscopic delineation of dental boundaries and internal structures, enhancing diagnostic accuracy, and clinical utility.

**Figure 3:**
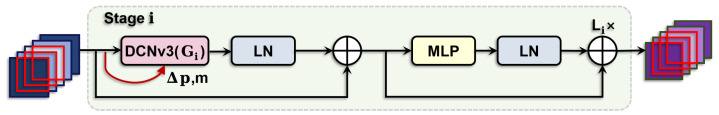
The schematic depiction of each hierarchical stage, composed of DCNv3, LN, and MLP, utilizes DCNv3 as its core operator for efficient feature extraction.

The lower part of Figure 2 depicts the Adaptive Deformable Pathway, utilizing DCNv3. This advanced convolution technique adjusts the shape and position of the convolutional kernels dynamically through learnable offsets and modulation factors, allowing the model to respond adaptively to local structural deformations and irregularities within dental X-ray images. The pathway initiates with a Stem module that performs successive convolutions, transforming the tri-channel input image into a feature map, subsequently processed through GELU activation functions and normalization layers. This treated feature map then progresses through four hierarchical stages of feature extraction, each stage comprising several base blocks focused on further refining the feature detail. As shown in Figure 3, each base block harnesses DCNv3 as its core operator for effective feature extraction, augmented by grouped convolution techniques to enhance the model’s expressive capacity. Incorporating DropPath [20] technology within these blocks serves to drop connections during training, mitigating overfitting randomly. Each convolutional operation is accompanied by LayerNorm normalization and GELU activation, ensuring data stability and introducing essential non-linearity. Selected blocks include feature scaling to accentuate critical features and enhance recognition capabilities. Following the comprehensive feature extraction across stages, the output feature maps of each stage are downsampled to expand the receptive field and reduce computational demands progressively, culminating in an integrated output sequence that encapsulates multi-scale features from granular to macroscopic levels. The computational process for each stage level can be defined as:

(1) G(f)=LN(MLP(LN(DCN(f))+f)+LN(DCN(f)))

(2) $${\mathrm{Stage}}^{\left(i+1\right)}=\mathrm{Iterate}\left(G,{\mathrm{Stage}}^{\left(i\right)},L_{i}\right)$$

where *G* represents each base block, $L_{i}$ denotes the number of base blocks in the *i*-th stage layer, and $\mathrm{Iterate}\;(G,{\mathrm{Stage}}^{\left(i\right)},L_{i})$ indicates the iteration of *G* across the ${\mathrm{Stage}}^{\left(i\right)}$ for $L_{i}$ times. DCN stands for Deformable Convolution, and LN for Layer Normalization.

The DCNv3, acting as a dynamic sparse convolution operator within this architecture, processes the input feature map *x* of dimension C×H×W. Each pixel $p_{i}$ in the output $DCN(p_{i})$ is determined by the weighted sum of multiple sampled points. These sampled points incorporate both position offsets $\Delta p_{k}$ and modulation factors $m_{k}$. Multiple aggregation groups are introduced, each handling a subset of the input feature map and possessing its own sampling offsets and modulation factors. The position offsets allow the convolution kernel to flexibly adjust its sampling locations to fit specific areas of the input features better, while the modulation factors are normalized across all sampled points via a softmax function, regulating the contribution of each sample point. The output for each pixel $p_{i}$ in the DCNv3 is defined as:

(3) $$DCN\left(p_{i}\right)=\sum_{g=1}^{G}\sum_{k=1}^{K}w_{g}m_{gk}x_{g}\left(p_{i}+p_{k}+\Delta p_{gk}\right)$$

where *G* represents the number of aggregation groups. *K* is the number of sampled points per group. $w_{g}$ denotes the position-independent projection weights for group *g*. $m_{gk}$ is the modulation factor for the *k* th sampled point in group *g*, normalized via a softmax function. $x_{g}$ is a slice of the input feature map corresponding to the group *g*. $\Delta p_{gk}$ represents the offset for the *k* th sampled point in group *g*.

**Figure 4:**
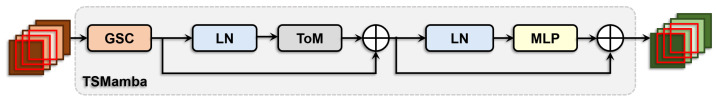
The schematic depiction of the TSMamba block involves GSC, ToM, LN, and MLP, collectively enhancing input feature processing and representation.

The upper part of Figure 2 showcases the parallel State Space Pathway, which handles spatial features of the image by iteratively updating the state to adapt to and recognize specific dental areas effectively within the dental X-ray images. This application of SSM, typically designed for capturing and modeling dynamic changes in time-series data, is innovatively applied in the spatial dimension for dental image segmentation. It focuses on global features and multi-scale modeling to pre-model dental structural dynamics, thereby improving the model’s focus on critical locations and ameliorating the challenges posed by the compression of three-dimensional structures into a two-dimensional representation typical of X-ray imaging. Within this encoder pathway, layers sequentially process the input through subsampling layers, GSC modules, Mamba layers, and MLP layers, ultimately outputting a processed feature sequence for use by the subsequent decoder. This pathway begins with a combination of a stem layer and multiple TSMamba blocks, aiming for efficient modeling of both multi-scale and global features. In the stem layer, deep convolution with a kernel size of 7 × 7, padding of 3, and stride of 2 extracts the initial scale feature from the input volume $I\in R^{C\times H\times W}$ to $z_{0}\in R^{48\times \frac{H}{2}\times \frac{W}{2}}$. Subsequently, $z_{0}$ is passed through each TSMamba block and its corresponding subsampling layer. As illustrated in Figure 4, the computational process for each TSMamba block can be defined as:

(4) $$\begin{array}{cc} f_{m}^{\left(l+1\right)}=\mathrm{MLP}( & \mathrm{LN}(\mathrm{ToM}\left(\mathrm{LN}\left(\mathrm{GSC}\left(f_{m}^{\left(l\right)}\right)\right)+\mathrm{GSC}\left(f_{m}^{\left(l\right)}\right)\right)\\ \; & +\mathrm{ToM}(\mathrm{LN}\left(\mathrm{GSC}\left(f_{m}^{\left(l\right)}\right)+\mathrm{GSC}\left(f_{m}^{\left(l\right)}\right)\right))) \end{array}$$

where GSC represents the Gate-Space Convolution module, ToM denotes the Tri-directional Mamba module, LN is Layer Normalization, and MLP stands for Multi-Layer Perceptron, utilized for enhanced feature representation.

The modified GSC module initially processes 2D features through two convolution blocks, one with a kernel size of 3 × 3 and the other with 1 × 1. Then, these two features undergo element-wise multiplication controlled by a gating mechanism. Subsequently, another convolution block further integrates the features while employing a residual connection to reuse the input feature. Its mathematical expression is:

(5) $$\mathrm{GSC}\left(f\right)=f+C_{3\times 3}\left(C_{3\times 3}\left(f\right)\cdot C_{1x1}\left(f\right)\right)$$

where $C_{3\times 3}$ and $C_{1\times 1}$ denote the corresponding size convolution operations. After processing by the GSC module, features are modeled for global information through the ToM module.

The ToM module within the TSMamba block effectively models high-dimensional global features by flattening 2D input features into three sequences for respective feature interaction, processed through Mamba layers, and then adding the processed sequences to form an integrated output feature. Its formula is:

(6) $$\mathrm{ToM}\left(f_{F},f_{R},f_{S}\right)=\delta \left(f_{F}\right)+\delta \left(f_{R}\right)+\delta \left(f_{S}\right)$$

where $f_{F},f_{R},f_{S}$ represent the forward, reverse, and slice-direction sequences, δ represent Mamba.

### 3.2. Cognitively Optimized Semantic Enhance Module (SEM)

**Figure 5:**
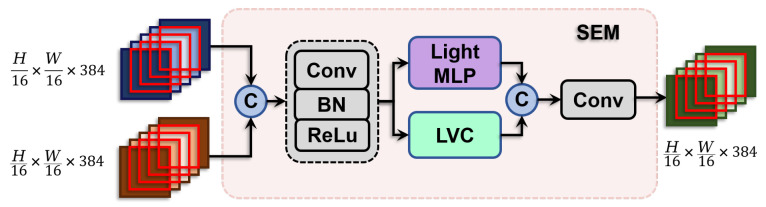
The schematic depiction of the SEM, which combines encoder outputs through concatenation, applies Conv, BN, and ReLU and then enhances features with the MLP and LVC. The MLP captures global dependencies, while the LVC focuses on local details.

In the present study, we propose a novel network module, the Cognitively Optimized Semantic Enhance Module, engineered to augment feature representation efficacy by synthesizing and enhancing outputs derived from two distinct pathways within the encoder, as illustrated in Figure 5. This module mitigates the complexities of amalgamating high-dimensional feature outputs from disparate sources. Concurrently, it ensures the refinement of features on the initial feature maps by implementing advanced smoothing techniques.

Initially, SEM fuses the high-dimensional outputs of the two encoder pathways by channel-wise concatenation, followed by a comprehensive feature processing initialized by a Stem block. This block incorporates a large convolutional kernel (7 × 7) and subsequent batch normalization and ReLU activation layers, setting the stage for advanced feature manipulation.

The concatenated feature output, represented as $F_{\mathrm{in}}$, is described by the equation:

(7) $$F_{\mathrm{in}}=\mathrm{CBR}\left(C_{\;at}\left(X,Y\right)\right)$$

where CBR denotes the sequence of Convolution (Conv), Batch Normalization (BN), and ReLU activation. *X* and *Y* represent the high-dimensional feature outputs from the preceding encoder stages. $C_{\;at}$ represents the channel-wise concatenation of the feature maps.

To further enhance the integrated features, SEM employs a Multi-Layer Perceptron (MLP) and a Learnable Vision Center mechanism (LVC) [18], which process the features before another concatenation phase to enrich global and local information processing. The MLP module focuses on capturing global dependencies across the entire image, while the LVC encodes local features, preserving and enhancing local details. The combination of these processes can be formulated as:

(8) $$SEM\left(X,Y\right)=C_{\;at}\left(\mathrm{MLP}\left(F_{\mathrm{in}}\right),\mathrm{LVC}\left(F_{\mathrm{in}}\right)\right)$$

where SEM(X,Y) denotes the final output from the SEM. The inputs $\mathrm{MLP}(F_{\mathrm{in}})$ and $\mathrm{LVC}(F_{\mathrm{in}})$ refer to the outputs from the MLP and vision center mechanism, respectively.

The Local Visual Codeword approach achieves cognitive optimization by preserving and enhancing local features. This is done by encoding them using a built-in visual dictionary, where each codeword represents a specific visual concept or pattern. The encoded features *F* are processed by combining the inherent codebook and scaling factors. The specific enhancement process of the LVC can be expressed with the following equation:

(9) $$\mathrm{LVC}\left(F_{\mathrm{in}}\right)=F_{\mathrm{in}}+F_{\mathrm{in}}\cdot \sigma \left({\mathrm{Conv}}_{1\times 1}\left(\sum_{k=1}^{K}\left(\sum_{i=1}^{N}\frac{e^{\alpha_{k}}\cdot {\left(x_{i}-\mu_{k}\right)}^{2}}{\sum_{j=1}^{K}e^{\alpha_{j}}\cdot {\left(x_{i}-\mu_{j}\right)}^{2}}\right)\right)\right)$$

where $F_{\mathrm{in}}$ represents the input features. σ is the Sigmoid activation function. $x_{i}$ is the *i*-th pixel point in the image, representing the feature vector at that location. $\mu_{k}$ is the k-th learnable visual codeword. $\alpha_{k}$ is the scaling factor for the *k*-th codeword. The term ${\left(x_{i}-\mu_{k}\right)}^{2}$ denotes the squared Euclidean distance between the *i*-th pixel point and the *k*-th visual codeword. *K* denotes the total number of visual centers or codewords involved in the process. ${\mathrm{Conv}}_{1\times 1}$ is a convolutional layer utilizing a 1 × 1 kernel to integrate the feature responses.

### 3.3. Hierarchical Convergence Decoder (HCD)

**Figure 6:**
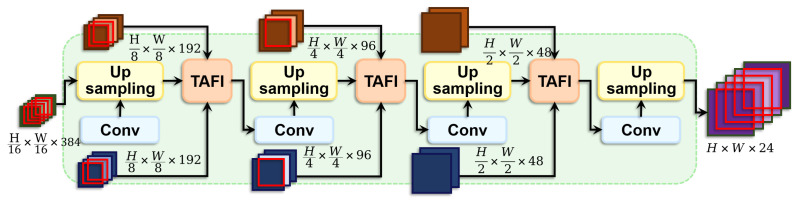
The schematic illustrates the HCD, incorporating a multi-layered decoder structure. Each tier combines convolutional and deconvolutional layers for feature enhancement and upsampling, and it is equipped with the TAFI designed specifically for feature fusion.

Traditional segmentation models often falter when segmenting 2D dental X-ray images due to the tooth’s complex structures and similarity in density and texture to adjacent tissues. These challenges hinder the effective integration of multi-scale information, which is crucial for accurate segmentation. To address this, we introduce a novel neural network module termed the Hierarchical Convergence Decoder (HCD), specifically designed to optimize hierarchical feature fusion, as illustrated in Figure 6.

The HCD module combines multi-dimensional hierarchical features extracted from two pathways of the CSDE and the high-dimensional features enhanced by the SEM. This strategy can preserve various types of feature information while balancing local and global information. The feature fusion strategy within HCD employs a multi-layered decoder architecture, executing through four specially designed resolution levels. Each level integrates convolutional and deconvolutional layers to refine features progressively and to upsample them, equipped with the Triplet Attentional Feature Integration (TAFI) module designed specifically for feature fusion. This module enables the dynamic integration of multi-level features from the encoder and the current processing stage at different decoding phases.

**Figure 7:**
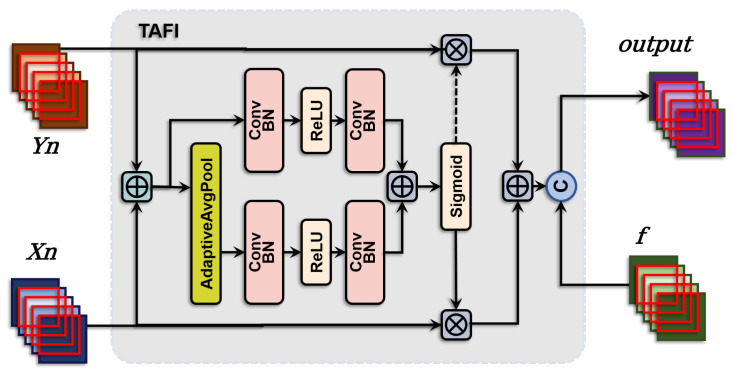
Schematic representation of the TAFI, which combines features from the encoder’s two pathways and uses local and global attention modules to emphasize important information.

As depicted in Figure 7, TAFI initially performs a dynamic fusion of skip connection features from two pathways of the encoder. This step employs an attention mechanism that emphasizes important information within the input features and suppresses less relevant parts, generating a highly representative intermediate feature. Subsequently, this intermediate feature is concatenated with pre-processed features to retain unique information from each feature type. The process is mathematically described as follows:

(10) $$\begin{array}{cc} \mathrm{output}=\; & C_{\;at}\left((X_{n}\cdot \sigma \left(\mathrm{LoAtt}\left(X_{n}+Y_{n}\right)+\mathrm{GlAtt}\left(X_{n}+Y_{n}\right)\right)\right.\\ \; & \left.+Y_{n}\cdot \left(1-\sigma \left(\mathrm{LoAtt}\left(X_{n}+Y_{n}\right)+\mathrm{GlAtt}\left(X_{n}+Y_{n}\right)\right)\right),f\right) \end{array}$$

where σ is the Sigmoid activation function, $X_{n}$ and $Y_{n}$ represent the features from the $n^{th}$ layer of the encoder output, *f* represent features from the upper floor of HCD, LoAtt and GlAtt are the local and global attention modules, respectively, with their operations defined as:

(11) LoAtt(x)=BN(Conv(ReLU(BN(Conv(x)))))

(12) GlAtt(x)=BN(Conv(ReLU(BN(Conv(AdaptiveAvgPool(x))))))

Through innovative structural design and hierarchical feature fusion strategies, HCD dynamically adjusts feature fusion strategies across different decoding stages using the TAFI module, effectively utilizing multi-scale information. Hierarchical feature fusion ensures detailed feature processing from coarse to fine, gradually refining global information and capturing details, making the model particularly suited for handling complex, multi-scale information.

## 4. Experiments and Results

### 4.1. Dataset

Our dataset for this study originates from the 2023 MICCAI Teeth Segmentation Challenge (https://tianchi.aliyun.com/competition/entrance/532086/information) on 1 May 2024, which was collected by Zhang Y et al. [21]. It comprises two-dimensional panoramic dental X-ray images, each with a resolution of 320 × 640 pixels. These X-ray images are three-channel images, and the labels are binary maps. All images are stored in PNG format. We utilized 2000 labelled images from the preliminary round as our training set. The labelled images in the training set were divided into a training subset and a validation subset in a 9:1 ratio, supporting the model’s training and validation processes. Moreover900, labelled images from the semifinals were employed as the test set to evaluate the model’s generalization capabilities and performance. This segmentation method aims to ensure the full utilization of data and provide a balanced and representative data environment to support the development and evaluation.

In addition to the MICCAI dataset, we further evaluated the generalizability of our model using the Tufts Dental Database. The Tufts Dental Database [22], a new X-ray panoramic radiography image dataset, consists of 1000 panoramic dental radiography images with expert labelling of abnormalities and teeth. The classification of radiography images was performed based on five different levels: anatomical location, peripheral characteristics, radiodensity, effects on the surrounding structure, and the abnormality category. This dataset provided an independent test set for our study, allowing us to assess the model’s performance on a diverse and clinically relevant set of images.

### 4.2. Evaluation Metrics

In the realm of image segmentation, a multifaceted evaluation strategy is crucial to comprehensively assess the performance of segmentation models. We used a variety of metrics: Dice Similarity Coefficient (DSC), 95% Hausdorff Distance (HD95), Intersection over Union (IoU), Accuracy, Kappa Coefficient, and Matthews Correlation Coefficient (MCC) [23]. Each metric offers distinct insights into the efficacy of the segmentation model under evaluation. By utilizing these metrics in conjunction, it is possible to evaluate the performance of segmentation models from multiple perspectives thoroughly. This approach not only considers the precision in the core and boundary areas of the model but also encompasses the overall predictive accuracy.

The Dice Similarity Coefficient is pivotal for gauging the model’s precision in identifying and delineating the target regions. It calculates the ratio of twice the area of overlap between the predicted and actual segments to the total number of pixels in both the predicted and actual segments:

(13) $$DSC=\frac{1}{N}\sum_{i=1}^{N}\frac{2\times |X_{pred,i}\cap Y_{true,i}|}{|X_{pred,i}\left|+\right|Y_{true,i}|}\times 100\%$$

where *N* is the total number of categories, $X_{pred,i}$ is the region of the $i^{th}$ predicted category, and $Y_{true,i}$ is the region of the $i^{th}$ true category.

The 95% Hausdorff Distance measures the maximum distance of the 95th percentile of the closest points between the model’s predicted boundaries and the actual boundaries, providing insight into the extremities of prediction error:

(14) $$\mathrm{HD}95=\max\left\{\max_{x\in X}\min_{y\in Y}d\left(x,y\right),\max_{y\in Y}\min_{x\in X}d\left(x,y\right)\right\},$$

where d(x,y) is the Euclidean distance between points *x* and *y*.

Intersection over Union evaluates the overall accuracy of the segmentation model by measuring the overlap between the predicted and actual segments:

(15) $$IoU=\frac{1}{N}\sum_{i=1}^{N}\frac{|X_{pred,i}\cap Y_{true,i}|}{|X_{pred,i}\cup Y_{true,i}|}\times 100\%$$

Accuracy offers a broad measure of the model’s performance across the entire image dataset, calculated by the ratio of correctly predicted pixels to the total pixels:

(16) $$\mathrm{Accuracy}=\frac{\mathrm{TP}+\mathrm{TN}}{\mathrm{TP}+\mathrm{TN}+\mathrm{FP}+\mathrm{FN}}.$$

The Kappa Coefficient assesses the degree of accuracy in prediction, accounting for the chance agreement. This metric is especially useful in imbalanced datasets:

(17) $$\kappa =\frac{p_{o}-p_{e}}{1-p_{e}},$$

where $p_{o}$ is the observed agreement, and $p_{e}$ is the expected agreement by chance.

Matthews Correlation Coefficient is a balanced measure that considers all four quadrants of the confusion matrix, ideal for evaluating models with imbalanced data classes:

(18) $$\mathrm{MCC}=\frac{\mathrm{TP}\times \mathrm{TN}-\mathrm{FP}\times \mathrm{FN}}{\sqrt{\left(\mathrm{TP}+\mathrm{FP})(\mathrm{TP}+\mathrm{FN})(\mathrm{TN}+\mathrm{FP})(\mathrm{TN}+\mathrm{FN}\right)}}.$$

By integrating these metrics, the evaluation framework not only highlights the segmentation model’s precision and accuracy in core and boundary areas but also ensures robust validation across various challenging scenarios. This comprehensive metric ensemble facilitates a deeper understanding of the model’s strengths and potential areas for improvement.

### 4.3. Implementation Details

Our framework is implemented based on PyTorch 2.0 and CUDA 11.7 and trained using an NVIDIA GeForce RTX 4090 GPU with 24 GB memory. We employ the Adam optimizer with an initial learning rate of 1 × 10^−5^ and train for 50 epochs. We utilize the ReduceLROnPlateau learning rate scheduling strategy to accelerate convergence and enhance model generalization. This strategy reduces the learning rate when the validation set loss does not decrease for several consecutive epochs, helping the model escape from local optima. Training is conducted with a batch size of 2. Data augmentation operations are applied to images and labels, including brightness adjustment, gamma transformation, Gaussian noise, scaling, mirror flipping, and spatial transformations [24]. These augmentations randomly alter the image attributes, thus improving the model’s generalization and robustness.

### 4.4. Loss Function Formulation

In a bid to optimize segmentation accuracy, our model utilized a composite loss function, combining Dice Loss and Cross-Entropy Loss. This hybrid loss function exploits the benefits of both loss types, enhancing training efficacy [25]. Specifically, Dice Loss is formulated as follows:

(19) $$\mathrm{Dice}\;\mathrm{Loss}=1-\frac{2\times |A\cap B|}{\left|A|+|B\right|}$$

where *A* and *B* represent the predicted and ground truth segmentation maps, respectively, prioritize the segmentation’s overlap accuracy, which is crucial in handling class imbalances. On the other hand, Cross-Entropy Loss focuses on pixel-wise classification accuracy, calculated by:

(20) $$\mathrm{CE}\;\mathrm{Loss}=-\frac{1}{N}\sum_{i=1}^{N}y_{i}\log\left({\hat{y}}_{i}\right)$$

where *N* is the total number of pixels, $y_{i}$ is the actual label, and ${\hat{y}}_{i}$ is the predicted probability. Integrating these two loss functions ensures a balanced focus on detailed pixel-level accuracy and overall segmentation quality, which is critical for the nuanced segmentation tasks required in dental X-ray imaging.

### 4.5. Comparison with State-of-the-Art Methods

Our network architecture, Deformable Convolution and Mamba Integration Network, integrates three innovative modules: Coalescent Structural Deformable Encoder, Cognitively-Optimized Semantic Enhance Module, and Hierarchical Convergence Decoder, specifically designed for the segmentation of dental X-ray images. To demonstrate our proposed method’s efficacy, we benchmarked it against 14 fully supervised mainstream medical segmentation methods, including OCNet, ICNet, and PSPNet, on the 2023 MICCAI Teeth Segmentation dataset.

**Figure 8:**
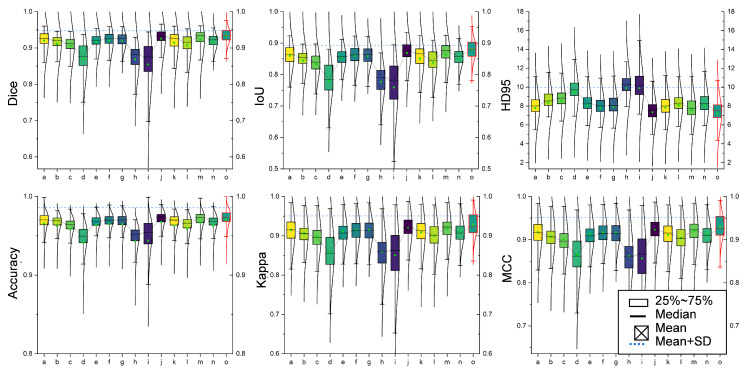
Box plot showcasing the evaluation metrics index from training results. On the x-axis, the models are labeled as follows: (a) ENet; (b) ICNet; (c) LEDNet; (d) OCNet; (e) PSPNet; (f) SegNet; (g) VM-UNet; (h) Attention U-Net; (i) R2U-Net; (j) UNet; (k) UNet++; (l) TransUNet; (m) Dense-UNet; (n) Mamba-UNet; (o) DeMambaNet (ours).

The comparative analysis demonstrates that our model surpasses OCNet, PSPNet, and UNet and their variants across multiple key performance indicators. As shown in Table 1, Table 2 and Figure 8, under uniform experimental conditions, our model achieved Dice and IoU scores of 93.38% and 87.81%, respectively, outperforming PSPNet’s scores of 91.97% and 85.27%. These results underscore our model’s heightened sensitivity in identifying dental regions, maintaining high segmentation accuracy even in images with high noise or low contrast. Additionally, our model excelled in accuracy and Kappa index, metrics that reflect correct pixel classification and consistency of classification performance, with scores of 97.45% and 91.78%, respectively, surpassing PSPNet’s scores of 96.86% and 89.99%. Regarding boundary precision, our model registered a 95% Hausdorff distance of 7.494, significantly lower than ICNet’s 8.700 and OCNet’s 9.838. This metric highlights our model’s superior edge localization, which is crucial for maintaining stable and accurate segmentation of dental and surrounding tissues in blurred boundary conditions, especially within complex overlapping areas.

**Figure 9:**
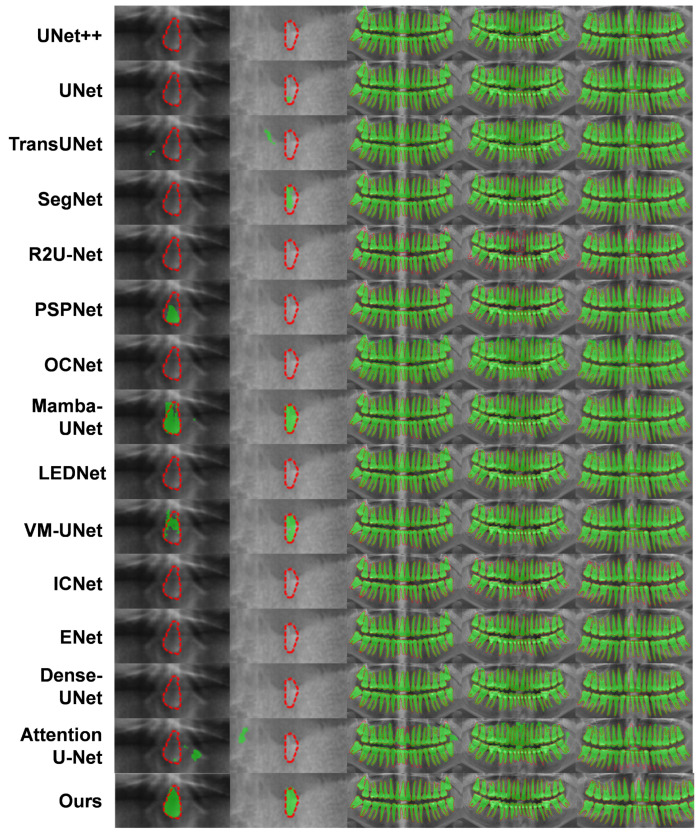
A few segmentation results of comparison between our proposed method and the existing state-of-the-art models. The segmentation result of the teeth is shown in green. The red dashed line represents the ground truth.

The performance of our network model is attributed to its three structural components. The CSDE module leverages the synergy of DCN and SSM, effectively adapting to local structural deformations and spatial features in dental images, thereby enhancing the model’s adaptability and precision over OCNet and PSPNet when dealing with overlapping and fine structures. The SEM facilitates efficient integration and enhancement of features from different encoders, providing superior detail resolution capabilities compared to PSPNet, which is crucial for accurately distinguishing dental from non-dental areas and aiding dental practitioners in interpreting X-ray images. The HCD module, through its multi-level feature fusion and detailed decoding process, further enhances the model’s resolution and detail expression. This meticulous hierarchical decoding strategy is particularly suited for high-resolution and structurally complex dental X-ray image segmentation. These results affirm our method’s capability in addressing the challenges of 2D dental X-ray image segmentation tasks. The robustness and adaptability of our model were also demonstrated through its ability to accurately segment normal dental structures and anomalies (e.g., missing or malformed teeth), as visually illustrated in Figure 9. This underlines the potential applicability and usefulness of our model in clinical settings.

## 5. Discussion

### 5.1. Ablation Experiment

**Figure 10:**
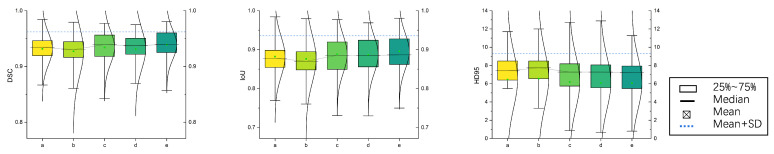
Box plot showcasing the evaluation metrics index of ablation experiments. On the x-axis, the models are labeled as follows: (a) w/o SSP; (b) w/o ADP; (c) w/o TAFI; (d) w/o SEM; (e) DeMambaNet (ours).

To evaluate the effectiveness of our proposed deep learning model in segmenting dental X-ray images, we conducted a series of ablation experiments, systematically removing key components of the model. The results, presented in Table 3 and Table 4 and Figure 10, not only confirm the importance of each model component but also reveal their individual contributions to the overall performance, particularly in addressing challenges, including compromised image fidelity, obscured delineation of structural boundaries, and the intricate anatomical structures of dental constituents such as pulp, enamel, and dentin.

The proposed architecture incorporates a Coalescent Structural Deformable Encoder, Semantic Enhance Module, and Hierarchical Convergence Decoder with the Triplet Attentional Feature Integration module. In the ablation studies, we evaluated the impact of these modules by individually removing each and observing the resultant performance variations.

The CSDE module integrates two specialized encoders: the Adaptive Deformable Pathway, based on DCN, and the State Space Pathway, based on SSM. This dual-pathway structure leverages the benefits of both approaches. DCN enables the encoder to adaptively modify the shape and position of the convolution kernels in response to local structural deformations in dental X-ray images, thereby enhancing the segmentation accuracy of complex dental structures such as tooth roots and overlapping areas. The State Space Pathway employs state space models to recognize specific dental features effectively and handle nonlinear characteristics and spatial variations within the image properties. Removal of the Adaptive Deformable Pathway from CSDE significantly degraded the model’s performance on detail, such as tooth edges and minor structures, reflected by an increased 95% Hausdorff distance. Additionally, when the State Space Pathway was removed, there was a slight decline in the model’s ability to process global information and maintain image quality, evidenced by minor drops in IOU and Dice coefficients.

The SEM module is designed to enhance and balance feature representation through feature fusion and enhancement. It utilizes an EVC mechanism to optimize the concatenation and enhancement of feature representations from the two output paths of the encoder, facilitating the effective integration of global and local features. The adoption of SEM resulted in improvements in several performance metrics, particularly noticeable in the 95% Hausdorff and IoU scores. The SEM’s use of multilayer perceptrons and a learnable visual center allows it to focus on the overall dental region while enhancing local details such as corner areas, which are critical in handling the complex edges where tooth roots overlap with adjacent tissues.

The HCD incorporates a multi-level feature fusion strategy and the TAFI module, which dynamically integrates multidimensional features from different layers (CSDE and SEM). The TAFI module enhances the representation power of the intermediate features by emphasizing important information and suppressing less relevant details. This coordinated integration of features from the dual-pathway encoders allows the model to handle complex multi-scale information more effectively. Comparative experiments show that our model, including the TAFI module, outperforms the baseline model without TAFI on all three key metrics: Dice Coefficient, IoU, and 95% Hausdorff distance. The Dice Coefficient and IoU improvements can be attributed to the innovative TAFI module’s ability to dynamically integrate multidimensional features, effectively balancing local and global information. Furthermore, a reduction in the 95% Hausdorff distance indicates a decrease in the maximum error in predicting tooth boundary positions, particularly beneficial in scenarios involving blurred boundaries of tooth roots.

These ablation experiments suggest the potential efficacy of each module within our model, indicating their roles in potentially improving the model’s segmentation accuracy and robustness when handling dental X-ray images.

### 5.2. Clinical Application

In dentistry, integrating deep learning techniques for segmenting 2D dental X-ray images holds promise for enhancing diagnostic accuracy and efficiency in treatment planning. These advanced technologies facilitate the segmentation of dental images, a crucial step in accurately diagnosing conditions such as caries and periodontal disease, and in devising personalized treatment plans. Moreover, by automating the segmentation process, these methods could significantly reduce the manual labor required, potentially improving workflow efficiency and increasing patient throughput in dental practices. Preliminary evidence supporting the efficacy of these technologies includes studies like those by Sheng et al. [44], which focus on optimizing segmentation in panoramic radiographs, and developments such as the STSN-Net architecture that proficiently segments and enumerates teeth, thereby offering potential improvements in clinical operations.

### 5.3. Clinical Implementation Challenges and Potential Limitations

The realization of high-precision tooth segmentation technology carries substantial significance in clinical settings, particularly in early diagnosis and treatment planning. However, translating this technology into a practical clinical tool entails several challenges. Although our DeMambaNet model excels in managing ambiguous boundaries in dental X-ray images, the complexity of real-world clinical environments may pose limitations. A key challenge is the dependency on high-quality data; discrepancies in data quality can adversely affect model performance, making consistent and high-standard image acquisition crucial for optimal clinical outcomes. When deciding how to implement this technology, medical professionals must consider various factors, from image capture to processing, including variations in imaging equipment and operational techniques.

Moreover, despite the superiority of our approach in handling images with high noise or low contrast boundaries compared to current clinical practices, the practical implementation of new technologies necessitates considerations of cost-effectiveness and personnel training. Additionally, accepting such technologies is critical and requires proper introduction and demonstration within medical teams to ensure seamless integration into everyday workflows.

Furthermore, although our method demonstrates theoretical and experimental advantages, extensive validation in clinical settings is required before widespread adoption. This includes multicenter clinical trials and long-term effectiveness assessments to ensure the technology’s reliability and stability.

## 6. Conclusions

In the segmentation of dental X-ray images, our study identifies several potential enhancements aimed at addressing a range of technical and medical challenges. These challenges include the diminished quality of images, the imprecise demarcation of structural boundaries, and the intricate anatomical features of dental components such as pulp, enamel, and dentin. Although these proposed improvements are designed to boost the performance of current methodologies, they may not completely overcome all the difficulties. Nonetheless, we believe that our initial investigations may pave the way for future research to further refine and adapt these techniques to more effectively tackle the issues outlined above.

Our novel 2D dental X-ray image segmentation network, Deformable Convolution and Mamba Integration Network, incorporates three groundbreaking modules: the Coalescent Structural Deformable Encoder, the cognitively-optimized Semantic Enhance Module, and the Hierarchical Convergence Decoder. The CSDE combines Deformable Convolution’s adaptive dynamic feature extraction capabilities with Mamba’s spatial feature handling prowess to dynamically simulate and capture the spatial structure characteristics of teeth. The SEM module integrates high-dimensional feature outputs through an efficient encoding strategy, enhancing and balancing feature representation of local details and global information. The HCD employs a layered feature fusion strategy to dynamically integrate and utilize multi-scale information, ensuring detailed processing from coarse to fine scales. Experimental comparisons with 14 baseline models demonstrated our model’s superior performance, achieving the DSC improvement of 0.95% over the best baseline and the HD95 of 7.494, lower than the best baseline score of 7.622. These results illustrate that our approach has advanced State of the Art in handling ambiguous boundaries in 2D dental X-ray image segmentation, especially in images with highly curved and overlapping intersections or high-noise, low-contrast boundaries, potentially transforming diagnostic and treatment workflows in dentistry.

Looking ahead, we plan to refine and optimize our methodology by implementing more flexible skip connection strategies and adjusting the decoder structure to effectively handle the integration of extensive feature information. We also intend to train our model on various pathological states of teeth, such as caries, dental calculus, and pulp disease, which requires the model to accurately segment and identify multiple pathological states to cope with complex clinical scenarios. This ongoing development and adaptation will ensure that our segmentation approach remains at the forefront of dental imaging technology, offering significant clinical benefits and enhancing the precision of dental disease diagnosis and treatment.

## Figures and Tables

**Table 1:** The comparison of our proposed method with state-of-the-art methods on the evaluation metrics in the 2023 MICCAI Teeth Segmentation Dataset. The best values for each metric are highlighted in red, while the second-best values are highlighted in blue.

Model	Dice (%) ↑	IoU (%) ↑	95 Hausdorff ↓	Accuracy (%) ↑	Kappa (%) ↑	MCC (%) ↑	GFLOPS ↓	Params (MB) ↓
UNet [26]	92.84±4.54	86.86±5.28	7.633±1.144	97.22±0.84	91.10±4.69	91.17±6.12	302.07	29.6
Dense-UNet [27]	92.55±4.94	86.38±5.61	7.920±1.145	97.16±0.91	90.77±5.11	90.80±6.60	497.41	17.51
UNet++ [28]	91.76±5.36	85.07±6.31	8.136±1.115	96.83±0.97	89.78±5.57	89.90±6.85	217.25	8.74
Mamba-UNet [29]	91.75±4.74	84.98±5.13	8.393±1.118	96.82±0.76	89.76±4.79	90.00±4.74	17.61	9.53
TransUNet [30]	90.87±6.57	83.67±6.94	8.423±1.041	96.49±0.87	88.67±6.60	89.05±6.50	147.51	64.72
Attention U-Net [31]	87.11±6.65	77.56±7.17	10.315±1.068	95.09±1.00	84.04±6.67	84.34±6.61	416.77	33.26
R2U-Net [32]	85.81±8.54	75.91±10.37	10.311±1.476	95.19±1.86	82.95±9.03	83.55±8.89	240.22	9.33
ENet [33]	92.33±4.77	85.98±5.21	8.101±1.073	97.03±0.82	90.46±4.87	90.50±6.42	**3.22**	**0.34**
ICNet [34]	91.42±4.84	84.43±5.33	8.700±0.982	96.80±0.77	89.42±4.90	89.32±6.42	57.88	26.98
LEDNet [35]	90.84±4.68	83.43±5.00	8.874±0.967	96.41±0.74	88.59±4.70	88.68±6.23	9.92	2.21
OCNet [36]	87.47±6.05	78.09±6.96	9.838±1.110	94.92±1.25	84.29±6.23	84.78±7.06	367.63	52.48
PSPNet [37]	91.97±3.63	85.27±4.17	8.361±0.977	96.86±0.68	90.00±3.68	90.03±5.55	288.95	46.5
SegNet [38]	92.33±3.73	85.91±4.44	8.071±1.000	97.00±0.72	90.45±3.84	90.51±5.65	251.24	28.08
VM-UNet [39]	92.24±3.90	85.75±4.66	8.200±1.094	96.98±0.78	90.34±4.02	90.47±4.94	33.41	26.16
DeMambaNet (ours)	93.38±4.80	87.81±5.30	7.494±1.165	97.45±0.84	91.78±4.93	91.98±4.87	216.24	41.25

**Table 2:** The comparison of our proposed method with methods on the evaluation metrics in the Tufts Dental Database. The best values for each metric are highlighted in red, while the second-best values are highlighted in blue.

Model	Backbone	Dice (%) ↑	IoU (%) ↑	Accuracy (%) ↑
UNet [26]	-	91.26	84.09	98.04
PSPNet [37]	ResNet18	91.49	85.66	94.76
DeepLabV3 [40]	ResNet18	91.87	86.02	94.91
DeepLabV3+ [41]	ResNet18	91.80	86.41	95.13
nnUNet [42]	**-**	90.86	86.11	94.91
CE-Net [43]	**-**	86.62	81.64	92.67
DeMambaNet (ours)	**-**	92.11	85.50	98.20

**Table 3:** We conducted ablation experiments on several key modules to assess their impact on overall performance. These modules include the State Space Pathway (SSP) and the Adaptive Deformable Pathway (ADP) in CSDE, SEM, and the TAFI module in HCD. We could quantify each module’s contribution by individually removing each module and observing performance changes. The best values for each metric are highlighted in red, while the second-best values are highlighted in blue. ↑indicates that higher values are better, ↓indicates that lower values are better.

SSP	ADP	TAFI	SEM	Dice (%) ↑	IoU (%) ↑	95 Hausdorff ↓	Accuracy (%) ↑	Kappa (%) ↑	MCC (%) ↑
\	√	√	√	92.37 ± 4.83	86.05 ± 5.34	8.01 ± 1.18	97.09 ± 0.84	90.55 ± 4.94	90.75 ± 4.89
√	\	√	√	91.91 ± 6.11	85.39 ± 6.43	8.14 ± 1.11	96.93 ± 0.76	89.99 ± 6.12	90.28 ± 5.94
√	√	\	√	92.96 ± 4.85	87.08 ± 5.45	7.66 ± 1.13	97.27 ± 0.85	91.25 ± 4.98	91.49 ± 4.90
√	√	√	\	93.24 ± 4.80	87.56 ± 5.33	7.55 ± 1.15	97.38 ± 0.84	91.60 ± 4.94	91.81 ± 4.88
√	√	√	√	93.38 ± 4.80	87.81 ± 5.30	7.49 ± 1.17	97.45 ± 0.84	91.78 ± 4.93	91.98 ± 4.87

**Table 4:** To demonstrate the contribution of the TAFI module in preserving diversity in feature representation across each layer of the decoder, we conducted an ablation study by selectively removing the TAFI from each layer individually. This approach allowed us to quantify the specific impact of TAFI at different stages of the decoding process, thereby highlighting its effectiveness in maintaining diverse feature representations. The best values for each metric are highlighted in red, while the second-best values are highlighted in blue. ↑indicates that higher values are better, ↓indicates that lower values are better.

TAFI1	TAFI2	TAFI3	Dice (%) ↑	IoU (%) ↑	95 Hausdorff ↓	Accuracy (%) ↑	Kappa (%) ↑	MCC (%) ↑
\	√	√	93.26 ± 5.68	87.67 ± 5.93	7.52 ± 1.14	97.45 ± 0.80	91.66 ± 5.74	91.84 ± 5.71
√	\	√	93.02 ± 4.72	87.16 ± 5.10	7.67 ± 1.09	97.31 ± 0.76	91.33 ± 4.81	91.54 ± 4.76
√	√	\	93.36 ± 4.76	87.76 ± 5.18	7.51 ± 1.16	97.46 ± 0.80	91.77 ± 4.86	91.94 ± 4.82
√	√	√	93.38 ± 4.80	87.81 ± 5.30	7.49 ± 1.17	97.45 ± 0.84	91.78 ± 4.93	91.98 ± 4.87

## Data Availability

The original data presented in the study are openly available at https://tianchi.aliyun.com/competition/entrance/532086/information (accessed on 14 May 2024).

## Abbreviations

The following abbreviations are used in this manuscript:

DeMambaNet	Deformable Convolution and Mamba Integration Network
CSDE	Coalescent Structural Deformable Encoder
SEM	Cognitively Optimized Semantic Enhancement Module
HCD	Hierarchical Convergence Decoder
TAFI	Triplet Attentional Feature Integration
SSP	State Space Pathway
ADP	Adaptive Deformable Pathway
DCN	Deformable Convolutional Networks
SSM	Statistical Shape Models
AFF	Attentional Feature Fusion
EVC	Efficient Vision Center
MLP	Multi-Layer Perceptron
LVC	Learnable Vision Center
DSC	Dice Similarity Coefficient
HD95	95% Hausdorff Distance
IoU	Intersection over Union
MCC	Matthews Correlation Coefficient
